# Idiopathic multicentric Castleman disease and associated autoimmune and autoinflammatory conditions: practical guidance for diagnosis

**DOI:** 10.1093/rheumatology/keac481

**Published:** 2022-08-23

**Authors:** Andrés González García, Julián Fernández-Martín, Ángel Robles Marhuenda

**Affiliations:** Systemic Autoimmune and Orphan Diseases Unit, Department of Internal Medicine, Hospital Universitario Ramón y Cajal, IRYCIS, Madrid, Spain; Internal Medicine Department, Hospital Álvaro Cunqueiro, University of Santiago de Compostela, Santiago de Compostela, Spain; Autoimmune Diseases Unit of the Internal Medicine Service, Hospital La Paz, Madrid, Spain

**Keywords:** multi-centric Castleman disease, diagnosis, differential, cytokine storm, autoimmune diseases, IL-6

## Abstract

Idiopathic multicentric Castleman disease (iMCD) is an infrequent and life-threatening disorder characterized by systemic inflammatory symptoms, generalized lymphadenopathy, polyclonal lymphocyte proliferation and organ dysfunction caused by a hyperinflammatory state. It accounts for one-third to one-half of all multicentric Castleman disease (MCD) cases. iMCD is often associated with autoimmune manifestations that may precede the iMCD diagnosis, be identified at the same time or follow it. In addition, iMCD may also coincide with a number of autoimmune diseases (such as psoriasis or myasthenia gravis) or autoinflammatory diseases (such as familial Mediterranean fever). Moreover, diverse inflammatory disorders, such as rheumatoid arthritis, systemic lupus erythematosus, adult-onset Still disease, systemic juvenile idiopathic arthritis, immunoglobulin (IgG4) related disease, or the recently described VEXAS syndrome, can present clinical features or lymphadenopathy with histopathological ‘Castleman-like’ findings compatible with those of iMCD. Given the iMCD clinical heterogeneity and the overlap with other autoimmune or autoinflammatory disorders, iMCD diagnosis can be challenging. In this review, we explore the overlap between iMCD and inflammatory diseases and provide practical guidance on iMCD diagnosis in order to avoid misdiagnosis and confusion with other autoimmune or autoinflammatory conditions.

Rheumatology key messages

- Idiopathic multicentric Castleman disease (iMCD) is an infrequent and life-threatening condition whose diagnosis is challenging.
- iMCD is often associated with autoimmune manifestations and may coincide with diverse autoimmune diseases.
- Inflammatory disorders that can mimic iMCD should be excluded before the diagnosis is established.

## Introduction

Castleman disease (CD) is a non-clonal lymphoproliferative disorder characterized by systemic inflammation comprising a series of conditions that share some overlapping clinicopathological manifestations. CD is classified as unicentric CD (UCD) or multicentric CD (MCD) [1]. A subtype of MCD is caused by the human herpesvirus-8 (HHV-8) and is known as HHV-8-MCD, whereas HHV-8-negative MCD cases are considered idiopathic [1]. Idiopathic MCD (iMCD) accounts for one-third to one-half of all MCD cases. It is an infrequent and life-threatening disorder characterized by systemic inflammatory symptoms, generalized lymphadenopathy, polyclonal lymphocyte proliferation, and multiple organ system dysfunction caused by a hyperinflammatory state [1]. The iMCD underlying pathogenesis involves dysregulated cytokine activity, often including IL-6, leading to systemic symptoms of inflammation and generalized lymphadenopathy [2].

CD, including iMCD, is often associated with autoimmune manifestations. This manifestations may precede the iMCD diagnosis, or may be identified at the same time or follow the iMCD diagnosis [3]. Autoantibodies including ANA, ds-DNA, anti-ENA, ANCA, anti-cardiolipin antibody or a positive Coombs may also be present in patients with iMCD without fulfilling inflammatory diseases criteria [3]. In addition, iMCD may coincide with a number of autoimmune diseases, such as psoriasis or myasthenia gravis [3, 4]. Autoinflammatory conditions, such as familial Mediterranean fever, have also been reported to occur with iMCD [5].

On the other hand, diverse autoimmune disorders, such as RA or SLE [1], or autoinflammatory diseases, such as adult-onset Still’s disease [6] can present clinical features or lymphadenopathy with histopathological ‘Castleman-like’ findings compatible with CD [3, 4, 7]. These suggest that autoimmune and autoinflammatory diseases share same features of pathophysiology with iMCD [3]. These disorders that can mimic iMCD should be excluded before the diagnosis of iMCD is established [1].

Both the iMCD clinical heterogeneity and the overlap with other autoimmune or autoinflammatory disorders pose challenges for the diagnosis of patients with iMCD. In this review, we aim to explore the overlap between iMCD and inflammatory diseases. In addition, we provide practical guidance on iMCD diagnosis in order to avoid misdiagnosis and confusion with other autoimmune or autoinflammatory conditions.

## Methods

A literature search was performed in PubMed using the following terms: Castleman disease; multi-centric Castleman disease; angiofollicular ganglionic hyperplasia; benign giant lymphoma; Castleman’s; angiofollicular mediastinal lymph node hyperplasia; idiopathic plasmacytic lymphadenopathy; dermatopathic lymphadenitis; lymphoid hyperplasia; lymphoid follicular hyperplasia; Kimura disease; diagnosis; differential diagnosis; diagnostic; autoimmune diseases; autoantibodies; autoimmune; mimicker. The search was launched on 1 November 2021. Original articles and reviews in English or Spanish published the previous three years were evaluated. Other articles of interest selected by the authors or cited by other articles were also assessed.

## CD classification

CD includes UCD and MCD. The latter is divided into iMCD, HHV-8-associated MCD (HHV-8-MCD) and POEMS-associated MCD. POEMS is an acronym that stands for polyneuropathy, organomegaly, endocrinopathy, monoclonal plasma cell disorder and skin changes. Patients with POEMS syndrome may present with CD-like histopathological findings, what is known as POEMS-associated MCD (POEMS-MCD) [8].

iMCD can be further subclassified into iMCD-TAFRO and iMCD-not otherwise specified (iMCD-NOS) [1, 8, 9]. The TAFRO syndrome corresponds to a subtype of iMCD featuring thrombocytopenia (T), anasarca (A), fever (F), reticulin fibrosis (R) and organomegaly (O) [10].

**Figure 1.**
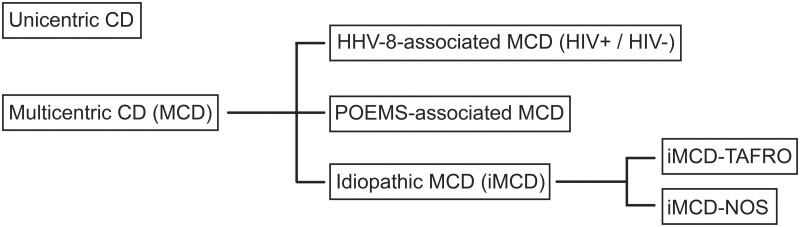
Castleman disease classification**. ** CD: Castleman disease; HHV-8: human herpesvirus-8; HIV: human immunodeficiency virus; iMCD: idiopathic multicentric Castleman disease; MCD: multicentric Castleman disease; NOS: not otherwise specified; POEMS: polyneuropathy, organomegaly, endocrinopathy, monoclonal plasma cell disorder and skin changes; TAFRO: thrombocytopenia, anasarca, reticulin fibrosis, renal dysfunction and organomegaly

CD classification is shown in Fig. 1 [1, 2, 8, 11–13].

## iMCD pathophysiology

The iMCD aetiology is still unclear. It has been suggested that iMCD involves polyclonal lymphoproliferation and hypercytokinaemia that are triggered by autoimmune or autoinflammatory mechanisms, paraneoplastic mechanisms associated to a clonal population, or an unidentified viral infection [13].

A hyperinflammatory state involving cytokines such as IL-6 is crucial for the pathogenesis of iMCD. Most patients have elevated levels of circulating cytokines and symptoms may improve with IL-6 inhibition or other forms of immunosuppression [13]. However, although increased amounts of interleukin 6 are common in patients with iMCD, subsets of symptomatic patients have normal or only slightly elevated levels of IL-6 [12]. In a case series, concentrations of IL-6 were raised in most (57/63) patients in whom IL-6 was measured [12].

IL-6, as a multifunctional or pleiotropic cytokine, may be responsible for autoimmune phenomena in iMCD by inducing the production of autoantibodies and the expansion of autoantibodies-producing CD5^+^ B-lymphocytes.

Besides, IL-6 could also dysregulate the cellular immune response by inducing proliferation and differentiation of T cells. In addition, increased NK cells observed in these patients indicate alterations in the innate immune system and may also participate in the development of autoimmunity [3].

The coexistence of CD, inflammatory disorders and autoantibodies, in the same patient, including quite specific autoantibodies such as anti-Sjögren-syndrome-related antigen A (SSA) or anti-centromere, may be explained because they likely share some pathophysiology features [3]. Excessive production of IL-6 and overactivation of IL-6 receptor (IL-6R) signalling may contribute to the development of other acute and chronic inflammatory disorders, including RA, GCA and several other autoimmune conditions [14].

Thus far, a clear genomic alteration causing iMCD has not been found. Increasing numbers of clonal alterations have been reported in patients with UCD and iMCD, where the underlying clonal neoplastic process could potentially lead to lymph node findings characteristic of CD and increased IL-6 in iMCD.

In a recent meta-analysis review, complex karyotypes in subsets of cases have been shown, as specific mutations in PDGFRB N666S in 10% of unicentric CD (UCD) and in NCOA4 L261F in 23% of idiopathic multicentric CD (iMCD) cases. Genes affecting chromatin organization and abnormalities in methylation are seen more commonly in iMCD, whereas abnormalities within the mitogen-activated protein kinase (MAPK) and interleukin signalling pathways are more frequent in UCD [15].

Interestingly, it has been suggested that autoinflammatory mechanisms involving a germ-line aberration in a gene of innate immunity may drive hypercytokinaemia [16]. Germ-line mutations involving important inflammatory genes, including IL-6 promoter polymorphisms, have been reported in iMCD [16–19]. In addition, iMCD can clinically resemble inflammatory diseases, such as SLE, adult-onset Still’s disease (AOSD), periodic fever syndromes, haemophagocytic syndromes or the VEXAS syndrome, which support this systemic inflammatory disease hypothesis [16].

## iMCD clinical presentation

All forms of MCD are characterized by a clinical presentation of systemic inflammatory symptoms, generalized lymphadenopathy (usually of relatively small volume), organ system dysfunction and laboratory abnormalities. The clinical spectrum of disease severity is very broad, ranging from minor symptoms to rapid, severe onset of symptoms that can result in life-threatening organ dysfunction. Symptoms usually progress rapidly; however, due to the rarity of the disease, clinical recognition is sometimes delayed. While asymptomatic UCD is common, asymptomatic MCD is not [13]. The inflammatory iMCD symptoms usually are intermittent, occurring in flares, whose precipitants are not clearly established. In more severe cases, once the inflammatory flare is established, it becomes self-sustained and may be life-threatening [13]. The main symptoms of iMCD are presented in Table 1 [13].

**Table 1.** iMCD main clinical manifestations

Fever
Weight loss
Generalized lymphadenopathy, usually of relatively small volume
Splenomegaly (which may be massive) and, in some cases, hepatomegaly
Anaemia (other cytopenias may be present)
Hypoalbuminaemia
Elevations in inflammatory markers
Anasarca
Intravascular depletion and renal impairment

Many clinical manifestations of the iMCD subtypes, despite some overlapping features, are different from those of iMCD-NOS and TAFRO [10]. iMCD-TAFRO comprises an aggressive clinical subtype of iMCD involving thrombocytopenia, anasarca, reticulin fibrosis, renal dysfunction and organomegaly [1]. Outcomes of this subgroup may be worse than the iMCD-NOS ones [10]. The presence of thrombocytopenia and the absence of hypergammaglobulinaemia are particularly suggestive of TAFRO syndrome in comparison with iMCD-NOS.

## iMCD histopathological features

Three distinct subtypes of CD can be distinguished based on lymph node pathology: hyaline-vascular, plasma cell or mixed cellularity type [10]. The term hyaline vascular was changed by a 2017 consensus to hypervascular when referring to iMCD [1]. However, the features present in the different histological subtypes are now considered to occur in a spectrum of pathologies rather than fitting into three easily definable groups. In this way, histological subtypes can be identified according to the predominance of hypervascular or plasmacellular features [1, 8].

This classification, although useful for histopathological diagnosis, does not have a well-defined clinical impact [1, 8]. UCD is more often associated with the hyaline-vascular subtype, whereas MCD, including iMCD, is more often associated with plasma cell and mixed subtypes [8, 10]. Lymph node iMCD-TAFRO pathology is characterized by the classic CD findings, but with higher frequency of mixed subtype and hypervascular features [1, 10].

The 2017 consensus for the diagnosis of iMCD established diagnostic criteria based on five histopathological features that could be found in all forms of CD: (i) regressed/atrophic germinal centres; (ii) follicular dendritic cells prominence; (iii) hypervascularity; (iv) polytypic plasmacytosis; and (v) hyperplastic germinal centres (Table 2) [1].

**Table 2.** Summary of consensus diagnostic criteria for iMCD

To make the diagnosis it is necessary that: Both major criteria are met (characteristic nodal histopathology and multicentric lymphadenopathy). At least two of the 11 minor criteria are met, with at least one laboratory abnormality. There is exclusion of autoimmune, infectious and malignant disorders that may mimic iMCD.				
Major criteria	Histopathology: Regressed/atrophic germinal centres (grade >2) with expanded mantle zones composed of concentric rings (‘onion skin’ appearance) Follicular dendritic cells prominence Hypervascularity with prominent endothelium in the interfollicular space and vessels penetrating into germinal centres (‘lollipop’ appearance) Sheet-like and polytypic plasmacytosis in the interfollicular space (grade >2) Presence of hyperplastic germinal centres		Enlarged lymph nodes: Enlarged lymph nodes (≥1 cm in short-axis diameter) in ≥2 lymph node stations	
Minor criteria	Clinical: 1. B symptoms 2. Hepatomegaly or splenomegaly 3. Fluid accumulation: oedema, anasarca, ascites, or pleural effusion 4. Lymphocytic interstitial pneumonitis 5. Eruptive cherry haemangiomatosis or violaceous papules		Laboratory: 6. Elevated CRP or ESR 7. Anaemia 8. Thrombocytopenia or thrombocytosis 9. Hypoalbuminaemia 10. Renal dysfunction or proteinuria 11. Polyclonal hypergammaglobulinaemia	
Selected additional features supportive of, but not required for diagnosis:				
Elevated IL-6, sIL-2R, VEGF, IgA, IgE, LDH and/or B2M Disorders that have been associated with iMCD: Paraneoplastic pemphigus Bronchiolitis obliterans organizing pneumonia Autoimmune cytopenias Polyneuropathy (without diagnosing POEMS) Glomerular nephropathy Inflammatory myofibroblastic tumour Reticulin fibrosis of bone marrow (particularly in patients with TAFRO syndrome)				
Exclusion criteria:				
Autoimmune or autoinflammatory diseases: Systemic lupus erythematosus Rheumatoid arthritis Adult-onset Still’s disease Juvenile idiopathic arthritis Autoimmune lymphoproliferative syndrome		Active or uncontrolled infection: HHV-8 HIV Toxoplasmosis EBV CMV		Malignancy: Lymphoma Multiple myeloma Plasmacytoma Follicular dendritic cell sarcoma POEMS syndrome

B2M: beta-2 microglobulin; CMV: cytomegalovirus; HHV-8: human herpesvirus-8; Ig: immunoglobulin; iMCD: idiopathic multicentric Castleman disease; LDH: lactate dehydrogenase; POEMS: polyneuropathy, organomegaly, endocrinopathy, monoclonal plasma cell disorder and skin changes; sIL-2R: soluble interleukin-2 receptor; TAFRO: thrombocytopenia, anasarca, reticulin fibrosis, renal dysfunction and organomegaly; VEB: Epstein–Barr virus; VEGF: Vascular endothelial growth factor.

Adapted from [1].

## iMCD diagnosis criteria

The proposed consensus diagnostic criteria include major and minor criteria. iMCD diagnosis requires both major criteria (characteristic lymph node histopathology and multicentric lymphadenopathy), at least two of the 11 minor criteria with at least one laboratory abnormality, and exclusion of infectious, malignant and autoimmune/autoinflammatory disorders that can mimic iMCD [1].

Consensus diagnostic criteria for iMCD are shown in Table 2 [1].

Diagnostic criteria specific for iMCD-TAFRO were proposed in 2016 by Iwaki *et al.* and include histopathological and clinical criteria [20, 21]. In these criteria, characteristic histopathological findings of lymph nodes are essential for diagnosis. However, lymph node biopsy from patients with TAFRO syndrome can be sometimes difficult or nearly impossible to obtain, due to anasarca, bleeding tendency or the small size or inaccessibility of the target lymph node. In addition, prompt diagnosis and initiation of treatment without delay are required to rescue these patients. Therefore, in 2019, Masaka *et al.* proposed new diagnostic criteria in order to include patients without histological lymph node confirmation [22].

However, in 2021, the 2016 diagnostic criteria of Iwaki *et al.* were updated incorporating the possibility of diagnosis without histological confirmation [23]. These authors classify TAFRO syndrome into three categories: (i) iMCD-TAFRO: TAFRO syndrome with lymph node histopathology compatible with iMCD; (ii) Possible iMCD-TAFRO: TAFRO syndrome without lymph node biopsy and without other co-morbidities (autoimmune/autoinflammatory, infectious or tumour pathologies); (iii) TAFRO without iMCD or other co-morbidities (TAFRO syndrome with lymph node histopathology not compatible with iMCD or other co-morbidities).

Therefore, these authors proposed that TAFRO syndrome is an entity that might be independent of MCD [23]. More time and studies are needed to establish the validity of this approach.

## Autoimmune manifestations and conditions associated to iMCD

iMCD may present with a wide range of autoimmune manifestations and also can be associated with features that are also found in systemic autoimmune diseases, such as glomerulopathy, pulmonary arterial hypertension or skin changes [4]. Autoimmunity-related symptoms, including arthritis and renal dysfunction with proteinuria, are more often observed in iMCD than in HHV-8-related MCD or UCD [10]. In accordance with iMCD diagnosis criteria, some of these autoimmune conditions support but are not required for iMCD diagnosis, including paraneoplastic pemphigus, bronchiolitis obliterans organizing pneumonia (BOOP), autoimmune cytopenias, polyneuropathy (without diagnosing POEMS), or glomerular nephropathy. However, paraneoplastic pemphigus and BOOP are more characteristic of UCD than iMCD.

In addition, iMCD may co-occur with several autoimmune or autoinflammatory conditions. Table 3 summarizes the main autoimmune manifestations and inflammatory conditions associated to iMCD [1, 3, 4, 8, 16, 24, 25].

**Table 3.** Autoimmune and autoinflammatory conditions associated with iMCD or mimicking iMCD clinical features or histopathology

Possible autoimmune manifestations of iMCD	Autoantibodies without a definitive autoimmune diagnosis (ANA, anti-platelet, anti-SSA, etc.) Autoimmune haemolytic anaemia Immune thrombocytopenic purpura Pulmonary arterial hypertension Sicca syndrome Glomerulopathy Paraneoplastic pemphigus Polyneuropathy (without diagnosing POEMS) Skin abnormalities including rash, hyperpigmentation or cherry haemangiomatosis Bronchiolitis obliterans organizing pneumonia Interstitial lung disease
Autoimmune/autoinflammatory diseases reported to co-occur with iMCD	Myasthenia gravis Psoriasis Amyloidosis Familial mediterranean fever Sarcoidosis Acquired factor VIII deficiency Pure red cell aplasia
Conditions that resemble histopathological iMCD findings	Haemophagocytic lymphohistiocytosis^a^ POEMS-associated MCD Autoimmune connective tissue diseases Systemic lupus erythematosus Rheumatoid arthritis Autoimmune lymphoproliferative syndrome IgG4-related disease Relapsing polychondritis VEXAS syndrome
Conditions that resemble clinical iMCD findings	Adult-onset Still’s disease Systemic juvenile idiopathic arthritis

^a^Haemophagocytic lymphohistiocytosis shares significant overlap with iMCD, but more data are needed to determine whether haemophagocytic lymphohistiocytosis should be excluded or considered an associated disease [1].

ANA: anti-nuclear antibodies; iMCD: idiopathic multicentric Castleman disease; POEMS: polyneuropathy, organomegaly, endocrinopathy, monoclonal plasma cell disorder and skin changes; SSA: Sjögren's-syndrome-related antigen A; VEXAS: vacuoles, E1 enzyme, X-linked, autoinflammatory, somatic.

Adapted from [1, 3, 4, 8, 20–22].

### Autoantibodies

Autoantibodies, ANA, anti-platelet and SSA may be found in up to 30% of iMCD patients [1, 12]. Other antibodies that can be found include: ds-DNA, ENA, ANCA or ACA [3].

The presence of autoantibodies may lead to a misdiagnosis of an inflammatory disease. IMCD could be suspected taking into account its clinical hallmarks [1]. However, it is often clinically unrecognized. Typically, iMCD is diagnosed when characteristic Castleman-like lymph node histopathological features are found in a node biopsy examination, HHV8 testing is negative, and other diseases that are known to cause these histopathological features are excluded [1]. Patients with autoantibodies, who do not meet the full criteria for an inflammatory condition and have pathologic features and other criteria consistent with CD are diagnosed as CD [1].

### Autoimmune cytopenias

Anaemia is a common symptom of iMCD. In the largest series, anaemia was reported in 79 of 91 patients in whom haemoglobin was measured [12]. It is often microcytic and consistent with anaemia of chronic inflammation [1, 26]. However, autoimmune haemolytic anaemia (AIHA) is a relatively frequent complication of MCD. It may be present in up to 30–40% of the patients and can be the initial presentation. Immune thrombocytopenia is less frequent but has been reported in 5% to 20% of MCD cases. The combination of AIHA and immune thrombocytopenia (Evans syndrome) has been reported but in the context of CD requires ruling out a diagnosis of autoimmune lymphoproliferative disorder [10].

Other causes of anaemia may be present in iMCD. Reticulin fibrosis of bone marrow is a characteristic feature in iMCD-TAFRO, although it can also be present in iMCD-NOS, and may result in anaemia [27]. Thrombotic microangiopathy (TMA) is a disorder that also presents with anaemia, together with thrombocytopenia, purpura and renal failure [28]. The classic conditions that cause TMA are haemolytic uraemic syndrome and thrombotic thrombocytopenic purpura, but iMCD-TAFRO is another possible cause [28].

Unexplained iron-deficiency anaemia has been rarely reported, particularly in paediatric UCD [29]. Chronic overproduction of IL-6 may be associated with inappropriate production of hepcidin, a peptide hormone secreted by the liver in response to iron loading and inflammation. Hepcidin blocks iron release from macrophages and hepatocytes and inhibits intestinal iron absorption [29–31].

### Peripheral neuropathy

Demyelinating peripheral neuropathy is frequently observed with CD. In fact, the presence of peripheral neuropathy is a feature that supports the iMCD diagnosis, although it is not necessary for the diagnosis [1]. The pathophysiology is unknown [10].

In UCD, peripheral neuropathy is less frequent and usually sensory in nature, whereas in the context of MCD, the neuropathy can be more severe and sensorimotor. Any clinical evidence of peripheral neuropathy requires careful evaluation of the diagnosis of POEMS syndrome [10]. In a patient with HHV-8 negative MCD and peripheral neuropathy, the following tests should be performed to rule out POEMS syndrome [8]: (i) A PET/CT scan to rule out sclerotic bone lesions (or review CT images); (ii) M protein in serum and urine protein electrophoresis; (iii) comprehensive endocrine testing (pituitary, thyroid, adrenal and gonadal axes); (iv) a bone marrow biopsy to rule out the presence of clonal plasma cells and megakaryocyte hyperplasia and atypia; and (v) pulmonary function tests and neurological assessments.

The severity of peripheral neuropathy is less in CD patients with peripheral neuropathy but not concurrent POEMS, followed by POEMS-MCD and worst in classic POEMS without MCD [8].

### Renal involvement

Renal dysfunction or proteinuria is frequently observed in MCD, mainly in the mixed or plasma cell subtype, with very heterogeneous clinical and histological findings [32]. Retrospective studies have reported renal abnormalities in up to 25% of MCD. Glomerular lesions, such as membranoproliferative glomerulonephritis (MPGN), secondary amyloidosis and interstitial nephritis are the most common renal pathology findings [10, 32–34]. Lesions of TMA can be observed in the presence of anti-ADAMTS13 antibodies [10].

In patients with TAFRO syndrome, TMA and MPGN-like lesions are the most common histological findings in a renal biopsy [35, 36]. Some patients may have both type of lesions [37, 38]. Abnormal production of IL-6 and vascular endothelial growth factor (VEGF) may explain the renal injury [36].

### Paraneoplastic pemphigus

Paraneoplastic pemphigus corresponds to the clinical presentation of pemphigus vulgaris in the context of a malignancy, mainly B-cell malignancy [10], but may also be associated with CD [39–44].

No specific histology of CD is associated with the existence of paraneoplastic pemphigus, but it is much more common in the context of UCD than in MCD [10].

In CD, the presence of mouth ulceration is highly suggestive of pemphigus and requires a careful skin and pulmonary evaluation [10]. Disease severity correlates directly with involvement of the lung. Pulmonary manifestations include dyspnoea, hypoxaemia, bronchiolitis obliterans and obstructive pulmonary ventilatory dysfunction. Autoantibodies targeting desmoplakin are often present [10].

There is no standardized treatment for paraneoplastic pemphigus. Systemic corticosteroids, azathioprine, mycophenolate mofetil, ciclosporin, intravenous and intralesional rituximab, cyclophosphamide, plasmapheresis and intravenous immunoglobulin have been used with variable results [39, 45–47].

## Inflammatory conditions that resemble clinical or histopathological MCD findings

The characteristic ‘Castleman-like’ histopathologic changes in MCD may be present in several inflammatory conditions (Table 3) [1, 10, 16, 48]. For example, 15%–30% enlarged lymph nodes from patients with SLE may display MCD-like histopathology [1]. POEMS-associated MCD, immunoglobulin G4-related disease (IgG4-RD) or the VEXAS syndrome are other conditions where histology findings may mimic those of iMCD and it is necessary to be taken into account. In addition, there are autoinflammatory diseases such as AOSD [6, 49] or systemic juvenile idiopathic arthritis (sJIA) [50] that may resemble the iMCD clinical features.

Any disorders that can mimic iMCD should be excluded before a definitive diagnosis of iMCD is established. The diagnostic evaluation should be based on the clinical presentation, and may require additional studies as indicated, and careful clinical correlation [1]. According to the iMCD diagnosis criteria, the following autoimmune or autoinflammatory conditions that share similar clinical or histopathological MCD findings have to be ruled out before an iMCD diagnosis is confirmed [1]: (i) SLE; (ii) RA; (iii) AOSD; (iv) juvenile idiopathic arthritis; and (v) autoimmune lymphoproliferative syndrome [51].

### Autoimmune connective tissue diseases: SLE and RA

Patients with SLE may present with generalized or localized lymphadenopathy. Even though lymphadenopathy is not included in the classification criteria for the disease, it is a commonly seen feature in SLE patients. Lymph node biopsy is performed more frequently in recent years to rule out malignancy [52], and the results may lead to an MCD misdiagnosis [53, 54]. In a study of lymph nodes from 21 patients with SLE, 6 out of 21 cases (29%) had histological features of CD [55]. In another study, 5 out of 33 SLE patients (15%) had features of CD [56]. On the other hand, iMCD patients may present lupus-like symptoms such as arthritis, cutaneous manifestations or renal disease which may cause a misdiagnosis [1].

Similarly, lymphadenopathy is frequently associated with RA. Reactive non-neoplastic tissue comprises the majority of the lymph node lesions. However, some of the histological disorders identified are compatible with MCD [57]. Similarly, as in iMCD, IL-6 dysregulation is critical in the pathogenesis of RA [14].

### Adult-onset Still’s disease and systemic juvenile idiopathic arthritis

AOSD is a systemic autoinflammatory disorder analogous to sJIA. Both diseases could be part of a spectrum, defined by the age of onset of the disease [58]. Clinically, AOSD [6, 49] and sJIA [50] may resemble iMCD, especially if the arthritis precedes other MCD manifestations. The diagnosis of these conditions is usually based on a thorough clinical evaluation and exclusion of other possible and more common disorders, together with criteria that can guide the diagnostic approach [58, 59]. Lymph node biopsy could help differentiate iMCD from these conditions [58].

### Autoimmune lymphoproliferative syndrome

Autoimmune lymphoproliferative syndrome (ALPS) is a rare non-malignant lymphoproliferative disorder whose precise aetiology has not yet been clarified in detail. The clinical presentations of ALPS includes lymphadenopathy, splenomegaly and autoimmune cytopenias which are caused by unregulated lymphocyte proliferation due to impaired T-cell apoptosis [51]. Its histopathological findings may mimic MCD histopathology [51]. The diagnostic criteria for ALPS were created by consensus in 1999, revised in 2010 and may help to differentiate this condition from iMCD [60].

### POEMS-associated MCD

POEMS syndrome is a paraneoplastic syndrome associated with clonal plasma cell neoplasms. POEMS stands for: peripheral neuropathy, organomegaly, endocrinopathy, monoclonal gammopathy, and skin changes [61]. Other clinical findings of POEMS syndrome include papilloedema, pleural effusions, ascites, sclerotic bone lesions and thrombocytosis [10, 61–63].

Classic POEMS syndrome is most often associated with osteosclerotic myeloma [8]. However, occasionally, patients with HHV-8 negative MCD are simultaneously diagnosed with POEMS syndrome; this co-existence is defined as POEMS-MCD. It is suspected that the same pathologic plasmatic cells causing the POEMS syndrome are also responsible for the concurrent MCD [8].

It has been agreed to distinguish POEMS-MCD from iMCD, because POEMS is associated with a monoclonal plasma cell disorder and has a different natural history and therapeutic approach than iMCD [1]. In fact, the presence of POEMS syndrome is an exclusion criterion for iMCD diagnosis [1].

Recently, the criteria for the diagnosis of POEMS syndrome have been updated. Mandatory major criteria include polyradiculoneuropathy and the presence of a monoclonal plasma cell-proliferative disorder [61]. Patients with CD and neuropathy without POEMS syndrome typically have a mild, painless and distal sensory neuropathy. In contrast, patients with POEMS syndrome with or without CD typically have painful sensorimotor neuropathy, most severe in those without CD [8, 62].

### IgG4-related disease

Another differential diagnosis of iMCD, especially of the plasma cell type, is IgG4-RD, which is a systemic inflammatory disorder characterized by sclerosing inflammation rich in IgG4-expressing plasma cells. This disease frequently affects the pancreas, salivary glands and lymph nodes, but it can involve almost any tissue [64–70]. Misdiagnosis may be due to the fact that iMCD patients may have an elevated serum IgG4 level, while some cases of IgG4-RD may show Castleman-like histopathology. Besides, both conditions present with systemic lymphadenopathy with extranodal involvement, and affected organs may overlap between the two conditions [2, 71].

In general, patients with IgG4-RD tend to be older than patients with iMCD [2]. Clinically, findings suggestive of iMCD are the presence of fever, high CRP, IL-6 and IgA levels and the absence of orbital, salivary gland and pancreatic involvement [71]. Atopic manifestations history is observed in almost 70% of the IgG4-RD cases but in <30% of patients with iMCD [66]. Serum IgG4 levels or absolute number of IgG4-positive cells in tissue are not useful for differentiating between the two conditions; the serum IgG4/IgG ratio and the ratio of IgG4/IgG-positive cells in tissue are more reliable differentiators [2].

Histologically, both conditions may be rich in plasma cells, but the plasma cells are often arranged in sheets in iMCD, whereas in IG4-RD are more commonly mixed with lymphocytes [2].

The diagnosis of IgG4-RD is based on the combined presence of the characteristic histopathological features and increased numbers of IgG4+ plasma cells demonstrated by immunohistochemistry. The critical histopathological features are a dense lymphoplasmacytic infiltrate, a storiform pattern of fibrosis and obliterative phlebitis [72, 73]. In addition, circulating plasmablasts/plasma cells expressing CD19^+^CD24^−^CD38^hi^ phenotypic markers are significantly elevated in IgG4-RD. This cell population might be a potentially useful biomarker for IgG4-RD diagnosis in the future [74].

On the other hand, exclusion criteria for IgG4‐RD have been proposed and include clinical findings (continuously elevated serum level of CRP, elevated serum level of IgA and elevated serum level of IgM) and pathological findings (sheet‐like proliferation pattern of mature plasma cells, high degree of haemosiderin deposition and neutrophilic infiltration) [70, 75].

A summary of the main differences between IgG4-RD and iMCD is shown in Table 4 [2, 55].

**Table 4.** Summary of different characteristics between IgG4-related disease and iMCD

	IgG4-RD	iMCD
Clinical features		
Atopic history (atopic dermatitis, allergic rhinitis, asthma)	Often	Rare
Exocrine gland involvement (lacrimal glands, salivary glands or pancreas)	Often	Rare
Lymph node involvement	Sometimes	Major criteria for diagnosis
Biomarkers		
CRP	Normal	High
Haemoglobin	Normal	Low
Platelet	Normal	High/low
Albumin	Normal	Low
IgG4: IgG ratio	High	Normal
IgA	Normal	High
IgM	Normal	High
IL-6	Normal	High
Histology		
Germinal centres expansion	Often	Sometimes
Mature plasma cells with sheet-like proliferation	Rare	Often
Haemosiderin deposition	Rare	Often
IgA+ cells	Rare	Abundant

IgG-4 RD: IgG4-related disease; iMCD: idiopathic multicentric Castleman disease. Adapted from [2, 55].

### VEXAS syndrome

Recently, a novel disorder named VEXAS syndrome was identified in patients with adult-onset inflammatory syndromes, often accompanied by myelodysplastic syndrome. VEXAS stands for vacuoles, E1 enzyme, X-linked, autoinflammatory and somatic [76]. This syndrome is a monogenic disease of adulthood caused by acquired mutations in UBA1, a gene encoding for the ubiquitin-activating enzyme 1, in haematopoietic progenitor cells [77].

Patients with VEXAS syndrome develop inflammatory and haematological symptoms including fever, cytopenia, dermatologic manifestations (including neutrophilic dermatosis and cutaneous vasculitis), pulmonary inflammation and chondritis [76, 77]. The VEXAS syndrome may overlap with myelodysplastic syndromes presenting with autoimmune disorders [78] and inflammatory syndromes [76]. Misdiagnosis of iMCD may also occur [25].

VEXAS syndrome can be diagnosed by bone marrow biopsy findings showing vacuolization of erythroid and myeloid precursors [79], biopsy of infiltrated skin lesions, that may show neutrophilic dermatosis with co-existing or exclusive leukocytoclastic vasculitis [80] and DNA sequencing [76].

## Conclusions

The working flowchart for diagnoses of iMCD may start by the first division into UCD and MCD. Then, MCD associated with HHV-8 infection, POEMS associated MCD and conditions mimicking MCD must be ruled out [24]. HHV-8 infection status may be determined through special staining of lymph nodes for latency-1;associated nuclear antigen-1 (LANA-1) and serology polymerase chain reaction test. Once the iMCD diagnose is made, TAFRO syndrome must be ruled out [24].

The diagnosis of iMCD is challenging and requires collaboration between clinicians and pathologists. The diagnosis of MCD is based on the clinical signs of systemic inflammation, serological tests and typical pathological features. It is important to exclude other diseases such as autoimmune diseases that have similar clinical manifestations and pathological findings.

## Data Availability

Data are available upon reasonable request by any qualified researchers who engage in rigorous, independent scientific research, and will be provided following review and approval of a research proposal and Statistical Analysis Plan (SAP) and execution of a Data Sharing Agreement (DSA). All data relevant to the study are included in the article.

## References

[1] Fajgenbaum DC , Uldrick TS, Bagg A et al International, evidence-based consensus diagnostic criteria for HHV-8–negative/idiopathic multicentric Castleman disease. Blood 2017;129:1646–57. 28087540 10.1182/blood-2016-10-746933 PMC5364342

[2] Zhou T , Wang HW, Pittaluga S, Jaffe ES. Multicentric Castleman disease and the evolution of the concept. Pathologica 2021;113:339–53. 34837092 10.32074/1591-951X-351 PMC8720411

[3] Sun DP , Chen WM, Wang L et al Clinical characteristics and immunological abnormalities of Castleman disease complicated with autoimmune diseases. J Cancer Res Clin Oncol 2021;147:2107–15. 33544201 10.1007/s00432-020-03494-2 PMC8164599

[4] Muskardin TW , Peterson BA, Molitor JA. Castleman disease and associated autoimmune disease. Curr Opin Rheumatol 2012;24:76–83. 22157415 10.1097/BOR.0b013e32834db525

[5] Endo Y , Koga T, Otaki H et al Idiopathic multicentric Castleman disease with novel heterozygous Ile729Met mutation in exon 10 of familial Mediterranean fever gene. Rheumatol 2021;60:445–50. 10.1093/rheumatology/keaa269 32830263

[6] Bianchi MM , Narváez J, Santo P et al Multicentric Castleman’s disease mimicking adult-onset Still’s disease. Joint Bone Spine 2009;76:304–7. 19213588 10.1016/j.jbspin.2008.10.005

[7] De Marchi G , De Vita S, Fabris M, Scott CA, Ferraccioli G. Systemic connective tissue disease complicated by Castleman’s disease: report of a case and review of the literature. Haematologica 2004;89:ECR03. 15075095

[8] Dispenzieri A , FajgenbaumDC. Overview of Castleman disease. Blood2020;135:1353–64.3210630210.1182/blood.2019000931

[9] Fujimoto S , Sakai T, Kawabata H et al Is TAFRO syndrome a subtype of idiopathic multicentric Castleman disease? Am J Hematol 2019;94:975–83. 31222819 10.1002/ajh.25554

[10] Szalat R , Munshi NC. Diagnosis of castleman disease. Hematol Oncol Clin North Am 2018;32:53–64. 29157619 10.1016/j.hoc.2017.09.005

[11] Srkalovic G , Marijanovic I, Srkalovic MB, Fajgenbaum DC. TAFRO syndrome: new subtype of idiopathic multicentric Castleman disease. Bosn J Basic Med Sci 2017;17:81–4. 28135567 10.17305/bjbms.2017.1930 PMC5474112

[12] Liu AY , Nabel CS, Finkelman BS et al Idiopathic multicentric Castleman’s disease: a systematic literature review. Lancet Haematol 2016;3:e163-175–e175. 27063975 10.1016/S2352-3026(16)00006-5

[13] Carbone A , Borok M, Damania B et al Castleman disease. Nat Rev Dis Primer 2021;7:84. 10.1038/s41572-021-00317-7 PMC9584164 34824298

[14] Kishimoto T , Kang S. IL-6 revisited: from rheumatoid arthritis to CAR T cell therapy and COVID-19. Annu Rev Immunol 2022;40:323–48. 35113729 10.1146/annurev-immunol-101220-023458

[15] Butzmann A , Kumar J, Sridhar K, Gollapudi S, Ohgami RS. A review of genetic abnormalities in unicentric and multicentric Castleman disease. Biology 2021;10:251. 33804823 10.3390/biology10040251 PMC8063830

[16] Fajgenbaum DC , van Rhee F, Nabel CS. HHV-8-negative, idiopathic multicentric Castleman disease: novel insights into biology, pathogenesis, and therapy. Blood 2014;123:2924–33. 24622327 10.1182/blood-2013-12-545087

[17] Stone K , Woods E, Szmania SM et al Interleukin-6 receptor polymorphism is prevalent in HIV-negative Castleman Disease and is associated with increased soluble interleukin-6 receptor levels. PloS One 2013;8:e54610. 23372742 10.1371/journal.pone.0054610 PMC3553080

[18] Koné-Paut I , Hentgen V, Guillaume-Czitrom S et al The clinical spectrum of 94 patients carrying a single mutated MEFV allele. Rheumatol 2009;48:840–2. 10.1093/rheumatology/kep121 19465590

[19] Wing A , Xu J, Meng W et al Transcriptome and unique cytokine microenvironment of Castleman disease. Mod Pathol 2022;35:451–61. 34686774 10.1038/s41379-021-00950-3 PMC9272352

[20] Iwaki N , Fajgenbaum DC, Nabel CS et al Clinicopathologic analysis of TAFRO syndrome demonstrates a distinct subtype of HHV-8-negative multicentric Castleman disease. Am J Hematol 2016;91:220–6. 26805758 10.1002/ajh.24242

[21] Igawa T , Sato Y. TAFRO Syndrome. Hematol Oncol Clin North Am 2018;32:107–18. 29157612 10.1016/j.hoc.2017.09.009

[22] Masaki Y , Kawabata H, Takai K et al 2019 Updated diagnostic criteria and disease severity classification for TAFRO syndrome. Int J Hematol 2020;111:155–8. 31782045 10.1007/s12185-019-02780-1

[23] Nishimura Y , Fajgenbaum DC, Pierson SK et al Validated international definition of the thrombocytopenia, anasarca, fever, reticulin fibrosis, renal insufficiency, and organomegaly clinical subtype (TAFRO) of idiopathic multicentric Castleman disease. Am J Hematol 2021;96:1241–52. 34265103 10.1002/ajh.26292 PMC9642098

[24] Wu YJ , Su KY. Updates on the diagnosis and management of multicentric Castleman disease. Tzu-Chi Med J 2021;33:22–8. 33505874 10.4103/tcmj.tcmj_15_20 PMC7821823

[25] Staels F , Betrains A, Woei-A-Jin FJSH et al Case report: VEXAS syndrome: from mild symptoms to life-threatening macrophage activation syndrome. Front Immunol 2021;12:678927. 34046042 10.3389/fimmu.2021.678927 PMC8147557

[26] Tabata S , Higuchi T, Tatsukawa S et al Idiopathic multicentric castleman disease with autoimmune hemolytic anemia and production of anti-drug antibody against tocilizumab. Intern Med 2019;58:3313–8. 31292389 10.2169/internalmedicine.2989-19 PMC6911740

[27] Belyaeva E , Rubenstein A, Pierson SK et al Bone marrow findings of idiopathic Multicentric Castleman disease: a histopathologic analysis and systematic literature review. Hematol Oncol 2022;40:191–201. 35104370 10.1002/hon.2969 PMC9547646

[28] Mizuno H , Sawa N, Watanabe S et al The clinical and histopathological feature of renal manifestation of TAFRO syndrome. Kidney Int Rep 2020;5:1172–9. 32775816 10.1016/j.ekir.2020.05.004 PMC7403508

[29] Arlet JB , Hermine O, Darnige L et al Iron-deficiency anemia in castleman disease: implication of the interleukin 6/hepcidin pathway. Pediatrics 2010;126:e1608–12. 21041280 10.1542/peds.2010-1123

[30] Nemeth E , Tuttle MS, Powelson J et al Hepcidin regulates cellular iron efflux by binding to ferroportin and inducing its internalization. Science 2004;306:2090–3. 15514116 10.1126/science.1104742

[31] Casper C , Chaturvedi S, Munshi N et al Analysis of inflammatory and anemia-related biomarkers in a randomized, double-blind, placebo-controlled study of siltuximab (anti-il6 monoclonal antibody) in patients with multicentric Castleman disease. Clin Cancer Res 2015;21:4294–304. 26124203 10.1158/1078-0432.CCR-15-0134

[32] Yuan XG , Hu W, Chen FF, Huang BF, Zhao XY. Renal complications of Castleman’s disease: report of two cases and analysis of 75 cases. Clin Exp Nephrol 2011;15:921–6. 21822592 10.1007/s10157-011-0499-9

[33] El Karoui K , Vuiblet V, Dion D et al Renal involvement in Castleman disease. Nephrol Dial Transplant 2011;26:599–609. 20656751 10.1093/ndt/gfq427

[34] Xu D , Lv J, Dong Y et al Renal involvement in a large cohort of Chinese patients with Castleman disease. Nephrol Dial Transplant 2012;27:iii119–125. 21602182 10.1093/ndt/gfr245

[35] Simeni Njonnou SR , Deuson J, Royer-Chardon C, Vandergheynst FA, Wilde VD. Unexplained cause of thrombocytopenia, fever, anasarca and hypothyroidism: TAFRO syndrome with thrombotic microangiopathy renal histology. BMJ Case Rep 2020;13:e234155. 10.1136/bcr-2019-234155 PMC7328895 32606113

[36] Tu KH , Fan PY, Chen TD et al TAFRO syndrome with renal thrombotic microangiopathy: insights into the molecular mechanism and treatment opportunities. Int J Mol Sci 2021;22:6286. 34208103 10.3390/ijms22126286 PMC8230834

[37] Leurs A , Gnemmi V, Lionet A et al Renal pathologic findings in TAFRO syndrome: is there a continuum between thrombotic microangiopathy and membranoproliferative glomerulonephritis? A case report and literature review. Front Immunol 2019;10. 10.3389/fimmu.2019.01489 PMC6609882 31316523

[38] Saito H , Tanaka K, Fujiwara M et al Pathological findings of progressive renal involvement in a patient with TAFRO syndrome. CEN Case Rep 2019;8:239–45. 31077056 10.1007/s13730-019-00400-9 PMC6820645

[39] Maruta CW , Miyamoto D, Aoki V et al Paraneoplastic pemphigus: a clinical, laboratorial, and therapeutic overview. An Bras Dermatol 2019;94:388–98. 10.1590/abd1806-4841.20199165 PMC7007015 31644609

[40] Wang L , Nong L, Li F et al Predominant stroma-rich feature in hyaline vascular variant of castleman disease is associated with paraneoplastic pemphigus. Am J Clin Pathol 2020;154:403–13. 32459333 10.1093/ajcp/aqaa053

[41] Han SP , Fu LS, Chen LJ. Masked pemphigus among pediatric patients with Castleman’s disease. Int J Rheum Dis 2019;22:121–31. 30411519 10.1111/1756-185X.13407

[42] Kim HJ , Han JH, Bang CH, Park KS et al Cutaneous disorders associated with Castleman’s disease. Acta Derm Venereol 2019;99:984–9. 31282978 10.2340/00015555-3253

[43] Kop EN , MacKenzie MA. Clinical images: Castleman disease and paraneoplastic pemphigus. CMAJ 2010;182:61. 19884299 10.1503/cmaj.081504 PMC2802608

[44] Ohzono A , Sogame R, Li X et al Clinical and immunological findings in 104 cases of paraneoplastic pemphigus. Br J Dermatol 2015;173:1447–52. 26358412 10.1111/bjd.14162

[45] Lim YL , Bohelay G, Hanakawa S, Musette P, Janela B. Autoimmune Pemphigus: latest advances and emerging therapies. Front Mol Biosci 2021;8:808536. 35187073 10.3389/fmolb.2021.808536 PMC8855930

[46] Viswanath V , Tare D, Patil PC. Successful use of intravenous and intralesional rituximab in paraneoplastic pemphigus with Castleman’s disease. Int J Dermatol 2021;60:e352–4–e354. 33720390 10.1111/ijd.15528

[47] Bin Waqar SH , Khan AA, Mohiuddin O, Rehan A. Paraneoplastic pemphigus with underlying Castleman’s disorder: a rare report with literature review. Cureus 2019;11:e5022. 31501721 10.7759/cureus.5022 PMC6721887

[48] Wu D , Lim MS, Jaffe ES. Pathology of Castleman disease. Hematol Oncol Clin North Am 2018;32:37–52. 29157618 10.1016/j.hoc.2017.09.004

[49] Lin SJ , Hsueh C, Chao HC. Localised hyaline vascular type of Castleman’s disease mimicking adult-onset Still’s disease. Clin Rheumatol 1999;18:485–7. 10638775 10.1007/s100670050143

[50] Osone S , Morimoto A, Tsutsui J et al Systemic juvenile idiopathic arthritis mimics multicentric Castleman’s disease. Clin Rheumatol 2003;22:484–6. 14677035 10.1007/s10067-003-0797-z

[51] Minemura H , Tanino Y, Ikeda K. Possible association of multicentric Castleman’s disease with autoimmune lymphoproliferative syndrome. BioRes Open Access 2018;7:47–51. 29682404 10.1089/biores.2017.0025 PMC5908426

[52] Demirkan FG , Doğan S, Kalyoncu Uçar A, Sönmez HE, Aktay Ayaz N. Systemic lupus erythematosus complicated with Castleman disease: a case-based review. Rheumatol Int 2021;41:475–9. 32797278 10.1007/s00296-020-04684-4

[53] Chasset F , Richez C, Martin T et al Rare diseases that mimic Systemic Lupus Erythematosus (Lupus mimickers). Joint Bone Spine 2019;86:165–71. 30837156 10.1016/j.jbspin.2018.10.007

[54] Wang L , Chen H, Shi J et al Castleman disease mimicking systemic lupus erythematosus: a case report. Medicine 2018;97:e12291. 30235674 10.1097/MD.0000000000012291 PMC6160051

[55] Kojima M , Nakamura S, Morishita Y et al Reactive follicular hyperplasia in the lymph node lesions from systemic lupus erythematosus patients: a clinicopathological and immunohistological study of 21 cases. Pathol Int 2000;50:304–12. 10849316 10.1046/j.1440-1827.2000.01052.x

[56] Kojima M , Motoori T, Asano S, Nakamura S. Histological diversity of reactive and atypical proliferative lymph node lesions in systemic lupus erythematosus patients. Pathol Res Pract 2007;203:423–31. 17540509 10.1016/j.prp.2007.03.002

[57] Kojima M , Motoori T, Nakamura S. Benign, atypical and malignant lymphoproliferative disorders in rheumatoid arthritis patients. Biomed Pharmacother 2006;60:663–72. 17064872 10.1016/j.biopha.2006.09.004

[58] Efthimiou P , Kontzias A, Hur P et al Adult-onset Still’s disease in focus: clinical manifestations, diagnosis, treatment, and unmet needs in the era of targeted therapies. Semin Arthritis Rheum 2021;51:858–74. 34175791 10.1016/j.semarthrit.2021.06.004

[59] Koker O , Demirkan FG, Cakmak F, Aktay Ayaz N. Performance of recent PRINTO criteria versus current ILAR criteria for systemic juvenile idiopathic arthritis: a single-centre experience. Mod Rheumatol 2021;roab115. 10.1093/mr/roab115 34850131

[60] Oliveira JB , Bleesing JJ, Dianzani U et al Revised diagnostic criteria and classification for the autoimmune lymphoproliferative syndrome (ALPS): report from the 2009 NIH International Workshop. Blood 2010;116:e35–40. 20538792 10.1182/blood-2010-04-280347 PMC2953894

[61] Dispenzieri A. POEMS syndrome: 2019 update on diagnosis, risk-stratification, and management. Am J Hematol 2019;94:812–27. 31012139 10.1002/ajh.25495

[62] Ali T , Qazilbash MH. POEMS syndrome: a multisystem clonal disorder. Eur J Haematol 2021;106:14–8. 32889731 10.1111/ejh.13514

[63] Albertí MA , Martinez-Yélamos S, Fernandez A et al 18F-FDG PET/CT in the evaluation of POEMS syndrome. Eur J Radiol 2010;76:180–2. 19581061 10.1016/j.ejrad.2009.06.004

[64] Carballo I , González-Quintela A, Sopeña B, Vidal C. Immunoglobulin G4–related disease: what an allergist should know. J Investig Allergol Clin Immunol 2021;31:212–27. 10.18176/jiaci.0633 32732179

[65] Chen LYC , Mattman A, Seidman MA, Carruthers MN. IgG4-related disease: what a hematologist needs to know. Haematologica 2019;104:444–55. 30705099 10.3324/haematol.2018.205526 PMC6395313

[66] Sasaki T , Akiyama M, Kaneko Y, Takeuchi T. Immunoglobulin G4–related disease and idiopathic multicentric Castleman’s disease: confusable immune-mediated disorders. Rheumatology 2022;61:490–501. 34363463 10.1093/rheumatology/keab634

[67] Wick MR , O'Malley DP. Lymphadenopathy associated with IgG4-related disease: diagnosis & differential diagnosis. Semin Diagn Pathol 2018;35:61–6. 29157939 10.1053/j.semdp.2017.11.006

[68] Otani K , Inoue D, Fujikura K et al Idiopathic multicentric Castleman’s disease: a clinicopathologic study in comparison with IgG4-related disease. Oncotarget 2018;9:6691–706. 29467920 10.18632/oncotarget.24068 PMC5805506

[69] Matsui S. IgG4-related respiratory disease. Mod Rheumatol 2019;29:251–6. 30474465 10.1080/14397595.2018.1548089

[70] Nishikori A , Nishimura MF, Nishimura Y et al Investigation of IgG4-positive cells in idiopathic multicentric Castleman disease and validation of the 2020 exclusion criteria for IgG4-related disease. Pathol Int 2022;72:43–52. 34762752 10.1111/pin.13185 PMC9299129

[71] Martín-Nares E , Hernández-Molina G, Baenas DF, Paira S. IgG4-related disease: mimickers and diagnostic pitfalls. J Clin Rheumatol 2022;28:e596–e604. 34538846 10.1097/RHU.0000000000001787

[72] Deshpande V , Zen Y, Chan JK et al Consensus statement on the pathology of IgG4-related disease. Mod Pathol 2012;25:1181–92. 22596100 10.1038/modpathol.2012.72

[73] Sato Y , Kojima M, Takata K et al Systemic IgG4-related lymphadenopathy: a clinical and pathologic comparison to multicentric Castleman’s disease. Mod Pathol 2009;22:589–99. 19270642 10.1038/modpathol.2009.17

[74] Lin W , Zhang P, Chen H et al Circulating plasmablasts/plasma cells: a potential biomarker for IgG4-related disease. Arthritis Res Ther 2017;19:25. 28183334 10.1186/s13075-017-1231-2 PMC5301376

[75] Satou A , Notohara K, Zen Y et al Clinicopathological differential diagnosis of IgG4‐related disease: a historical overview and a proposal of the criteria for excluding mimickers of IgG4‐related disease. Pathol Int 2020;70:391–402. 32314497 10.1111/pin.12932

[76] Beck DB , Ferrada MA, Sikora KA et al Somatic mutations in *UBA1* and severe adult-onset autoinflammatory disease. N Engl J Med 2020;383:2628–38. 33108101 10.1056/NEJMoa2026834 PMC7847551

[77] Grayson PC , Patel BA, Young NS. VEXAS syndrome. Blood 2021;137:3591–4. 33971000 10.1182/blood.2021011455 PMC8462403

[78] Huang H , Zhang W, Cai W et al VEXAS syndrome in myelodysplastic syndrome with autoimmune disorder. Exp Hematol Oncol 2021;10:23. 33741056 10.1186/s40164-021-00217-2 PMC7976711

[79] Patel N , Dulau-Florea A, Calvo KR. Characteristic bone marrow findings in patients with UBA1 somatic mutations and VEXAS syndrome. Semin Hematol 2021;58:204–11. 34802541 10.1053/j.seminhematol.2021.10.007

[80] Sterling D , Duncan M, Philippidou M et al VEXAS syndrome (vacuoles, E1 enzyme, X-linked, autoinflammatory, somatic) for the dermatologist. J Am Acad Dermatol 2022;S0190-9622(22)00181-5. 10.1016/j.jaad.2022.01.042 35121074

